# An update on the interaction between COVID-19, vaccines, and diabetic kidney disease

**DOI:** 10.3389/fimmu.2022.999534

**Published:** 2022-10-20

**Authors:** Yang Yang, Shubiao Zou, Gaosi Xu

**Affiliations:** ^1^ Department of Nephrology, The Second Affiliated Hospital of Nanchang University, Nanchang, China; ^2^ Department of Blood Transfusion, The Second Affiliated Hospital of Nanchang University, Nanchang, China

**Keywords:** COVID-19, SARS-CoV-2, diabetic kidney disease, ACE2, DPP4, NRP-1, vaccination

## Abstract

Up to now, coronavirus disease 2019 (COVID-19) is still affecting worldwide due to its highly infectious nature anrapid spread. Diabetic kidney disease (DKD) is an independent risk factor for severe COVID-19 outcomes, and they have a certain correlation in some aspects. Particularly, the activated renin–angiotensin–aldosterone system, chronic inflammation, endothelial dysfunction, and hypercoagulation state play an important role in the underlying mechanism linking COVID-19 to DKD. The dipeptidyl peptidase-4 inhibitor is considered a potential therapy for COVID-19 and has similarly shown organ protection in DKD. In addition, neuropilin-1 as an alternative pathway for angiotensin-converting enzyme 2 also contributes to severe acute respiratory syndrome coronavirus 2 entering the host cells, and its decreased expression can affect podocyte migration and adhesion. Here, we review the pathogenesis and current evidence of the interaction of DKD and COVID-19, as well as focus on elevated blood glucose following vaccination and its possible mechanism. Grasping the pathophysiology of DKD patients with COVID-19 is of great clinical significance for the formulation of therapeutic strategies.

## Introduction

The outbreak of the coronavirus disease 2019 (COVID-19) in Wuhan, China, in December 2019 is undoubtedly a serious blow to global health security and economic development. Several countries experienced a second or third wave, especially after the emergence of mutant viruses. This pandemic, caused by severe acute respiratory syndrome coronavirus 2 (SARS-CoV-2), enters host cells by binding to the angiotensin-converting enzyme 2 (ACE2) *via* a spike protein on its surface (1), initially presenting with fever, cough, and fatigue; as the condition progresses, hypoxemia, respiratory failure, and even death may occur (2). Meanwhile, the presence of comorbidities increases the risk of SARS-CoV-2 infection, including hypertension, obesity, and diabetes (3, 4).

Diabetic kidney disease (DKD) is one of the most common microvascular complications of both types of diabetes. The increasing prevalence of diabetes poses a major threat to DKD, especially an increase in type 2 diabetes (T2DM), and DKD is the most common cause of end-stage renal disease (5). During this pandemic, a study reported that DKD patients infected with SARS-CoV-2 require a longer hospital stay to recover and has the greatest impact on patients’ prognosis (6). Leon-Abarca et al. reported that patients with DKD are twice as likely to have COVID-19 pneumonia as patients with chronic kidney disease and have higher intubation rates and case-fatality rates, which may be associated with pro-inflammatory state and immune dysfunction (7). Notably, among the patients with maintenance hemodialysis (HD) with COVID-19, more than half of them were combined with DKD and they are more likely to be admitted to intensive care units (ICUs) and die (8). DKD patients are increasingly being appreciated as an infectable group for SARS-CoV-2, which is more prone to progress to a severe disease.

Hyperglycemia is the initial cause of the development of DKD; it can activate aldose reductase, protein kinase C, and advanced glycation end product (AGE) pathway, thus inducing the cell signal transduction network, resulting in inflammation response and cell damage. However, it has been reported that SARS-CoV-2 can infect pancreatic β-cells, leading to an imbalance in insulin homeostasis and apoptosis (9). Further research found that the concentrations of glucose, insulin, and insulin resistance were significantly increased in severe COVID-19 patients than in healthy people, resulting in increased oxidative and nitrosative stress (10), which may make it more difficult to treat and have serious consequences. Khunti et al. summarized the study of new-onset diabetes in COVID-19 (11). Thus, the disorder of glucose metabolism may cause a vicious circle in patients with DKD complicated with COVID-19.

Although the exact complex pathophysiology mechanism between COVID-19 and DKD is still being explored, the relationship between them seems to be traceable. In this review, we focus on DKD complicated with COVID-19 patients. We retrospect the pathogenesis of COVID-19 and DKD and put the spot on their correlation. Furthermore, we are also concerned about elevated blood glucose after COVID-19 vaccination.

## Clinical characteristics of DKD complicated with COVID-19 patients

In this review, we performed a literature search through the electronic database, including Medline/PubMed, EMBASE, and Web of Science before 20 July 2022, using the following keywords: (“diabetic kidney disease” OR “diabetic nephropathy” OR “diabetic glomerulosclerosis”) AND (“COVID-19” OR “2019-nCoV” OR “novel corona virus” OR “SARS-CoV-2” OR “coronavirus”).

We found 11 cases of DKD complicated with COVID-19 (12–19) (**Table 1**), who were hospitalized with COVID-19. In the literature, six of them were men and two cases reported a history of contact with COVID-19 patients, four patients required endotracheal intubation, and two patients died. Fever and cough were the most common clinical symptoms. After receiving timely treatment, most of the patients’ biochemical indexes gradually improved and were finally discharged from the hospital. Among the nine patients treated with HD, we found that two patients did not need HD before admission but underwent HD after admission, and one patient did not recover from HD.

**Table 1:** Summary cases of DKD complicated with COVID-19 patients.

Case	Authors	Age/sex	Country	Pre-hospital symptoms	Laboratory test after admission	Chest computed tomography	Treatments	Outcomes
1	Abe et al. (12)	60/M	Japan	Cough, slight fever, fatigue	Cr: 10.04 mg/dl D-dimer: 3.2 µg/ml CRP: ≈6 mg/dl Lymphocyte count: 530 × 10^6^/l	Bilateral and peripheral GGO	Peramivir, favipiravir, HD, tocilizumab, immunoglobulin, intubation	CRP decreased Continue to HD after discharged
2	Abe et al. (12)	68/F	Japan	Fever, cough, diarrhea	Cr: 4.69 mg/dl D-dimer: 9.7 µg/ml CRP: ≈13 mg/dl Lymphocyte count: 520 × 10^6^/l	Bilateral and peripheral GGO	Peramivir, favipiravir, tocilizumab, immunoglobulin, HD, intubation	CRP decreased Improved, discharged
3	Bishawi et al. (13)	32/F	Qatar	Cough, fever, body ache	Cr: 10.08 mg/dl	Right lower and mid GGO	Remdesivir, dexamethasone, HD	Cr fell to 3.85 mg/dl HD is not required after discharged
4	Bishawi et al. (13)	88/M	Qatar	Cough, fever	Cr: 1.39 mg/dl	Bilateral lower infiltrates and peripheries faint GGO	Remdesivir, dexamethasone, tocilizumab	Cr fell to 1.43 mg/dl Improved, discharged
5	Hertanto et al. (14)	59/F	Indonesia	Fever, cough, dyspnea, leg pain	Cr: 1.5 mg/dl D-dimer: 9.74 µg/ml CRP: 22.5 mg/dl	NA	Amputation, thrombectomy	Cr fell to 1.30 mg/dl Improved, discharged
6	Hirai et al. (15)	72/M	Japan	Fever, cough	NA	Left lower lobe consolidation	Favipiravir, HD, intubation	Died
7	Koshi et al. (16)	52/F	Japan	Fever and have a history of contact with a COVID-19 patient	D-dimer:1.06 µg/ml CRP: 3.91 mg/dl	Double lung lower lobe GGO	Favipiravir, HD	CRP decreased Improved, discharged
8	Kuroki et al. (17)	69/M	Japan	Fever, cough, dyspnea	CRP: 15.2 mg/dl Lymphocyte count: 1,010 × 10^6^/l	Bilateral multiple consolidation, GGO, and pleural effusion	Hydroxychloroquine, HD	CRP decreased Improved, discharged
9	Kuroki et al. (17)	72/F	Japan	Fever, dyspnea	Lymphocyte count: 430 × 10^6^/l	NA	Hydroxychloroquine, HD	Improved, discharged
10	Tang et al. (18)	50/M	China	Non-productive cough	CRP: 4.01 mg/dl Lymphocyte count: 530 × 10^6^/l	Bilateral multiple GGO	Moxifloxacin, lopinavir/ritonavir, HD	Improved Discharged
11	Yuan et al. (19)	49/M	China	Intermittent cough and have a history of contact with a COVID-19 patient	NA	A small cotton-wool spot in the right lobe of the right lung	Lopinavir, ritonavir, interferon alfa-2b, HD, intubation	Died

COVID-19, coronavirus disease 2019; Cr, creatinine; CRP, C-reactive protein; DKD, diabetic kidney disease; F, female; GGO, ground-glass opacities; HD, hemodialysis; M, male; ICU, intensive care unit; NA, not available.

In the literature, four patients had reported lymphocytic counts decreased and three cases reported cytokine storm (12, 13). Similar laboratory results show progressive reduction of lymphocytes in COVID-19 patients; among the various cytokine changes, interleukin-6 (IL-6) and IL-8 were the most significant (20). Elevated cytokines and chemokines, known as cytokine storms, further lead to dysregulation of the immune response, which are a feature of severe COVID-19 and are thought to be a major determinant of disease progression (2, 21). Research found that plasma concentrations of IL-2, IL-7, IL-10, and tumor necrosis factor- α (TNF-α) of COVID-19 patients admitted to the ICU were higher than those of non-ICU patients (2).

Furthermore, four cases of elevated D-dimer levels have been reported in the literature (12, 14, 16). D-dimer is a fibrin degradation product, and the increase in this level indicates that a hypercoagulable state *in vivo* exists that may be associated with subsequent thrombosis (22). Sixteen percent of COVID-19 patients hospitalized in the New York Health system reported thrombotic events, and the level of d-dimer at the time of visit was independently associated with thrombotic events (23). Li et al. indicated that higher levels of D-dimer on admission were associated with increased odds of death and significantly elevated in patients with diabetes (24); thus, the use of anticoagulants also needs to attract the attention of clinicians.

## DKD and COVID-19

### The role of ACE2

Renin–angiotensin–aldosterone system (RAAS) activation is a central mechanism in the pathogenesis of DKD. ACE2 is considered a new therapeutic strategy for DKD due to its renoprotective effect by converting angiotensin II (Ang II) to angiotensin 1–7 (Ang 1–7), which acts as a vasodilator, anti-inflammatory, anti-fibrotic, and anti-proliferation agent *via* its specific Mas receptor to counteract the side effects of Ang II (25, 26). This fact is well established by the evidence of akita mice whose blood pressure was reduced, elevated protein kinase C level was attenuated, and glomerular hypertrophy was relieved after injection with ACE2 (27). Mizuiri et al. revealed that the high ACE/ACE2 ratio in DKD patients may lead to a decrease in the glomerular filtration rate and renal injury (28). Moreover, Ang 1-7 *via* Mas protein stimulates the production of nitric oxide (NO) in platelets to exert an antithrombotic role, and this effect is inhibited in Mas knockout mice who exhibit shorter bleeding times (29).

Similarly to SARS, SARS-CoV-2 enters host cells precisely through ACE2 and, because of its stronger affinity to ACE2, leads to a widespread infection worldwide (1). SARS-CoV-2 enters kidney cells and activate immune response to stimulate the production of the cytokine factor. The involvement of the immune system and inflammation in the pathogenesis of DKD has been reported (30, 31). DKD-induced chronic inflammation in combination with the inflammatory response by SARS-CoV-2 results in cytokine storm, which in turn exacerbates renal injury. Sultan et al. demonstrated that cytokine storm caused by the high expression of ACE2 in COVID-19 patients may be one of the causes of renal dysfunction (32). Interestingly, Menon and colleagues compared DKD ACE2+ characterized genes with the published SARS-CoV-2 genes and found that both two genes significantly overlap and nearly 30% of genes are functionally aligned with key processes of viral infection and immune response (33). It means that some gene expression procedures of DKD can interact with the virus infection to some extent. In addition, Gilbert et al.’s biopsy of 49 DKD patients and 12 healthy living kidney specimens showed that the ACE2 mRNA expression in the former was twice as high as that in the latter (34), implying that they are more vulnerable to being infected with COVID-19 and impose a double burden on the kidneys.

Notably, not only does ACE2 contribute to SARS-CoV-2 entering cells but also internalization decreases its expression on the cell membrane. This results in the accumulation of Ang II, which increases aldosterone secretion, resulting in subsequent hypokalemia, which inhibits insulin secretion (35) and exerts vasoconstriction, oxidative stress, inflammation, fibrosis, and pulmonary edema by combination with the AT1 receptor (AT1R). It has been proved that Ang II combined with AT1R increases pulmonary capillary permeability, leading to increased pulmonary edema and lung damage (21). Exogenous injection of recombinant human ACE2 protein reduces the extent of acute lung injury in acid-treated ACE2 knockout mice (36), showing that ACE2 has a protective effect on lungs. Taken together, downregulation of the ACE2/Ang 1-7/Mas receptor axis and amplification of the ACE/Ang II/AT1R axis are harmful to DKD complicated with COVID-19 patients.

### Endothelial injury and thrombosis

Albuminuria is one of the earliest detectable abnormalities indicators of DKD. Thirty years ago, Deckert et al. hypothesized that proteinuria was a reflection of extensive microvascular injury (37) and was supported in subsequent results (38). Chronic hyperglycemia, reactive oxygen species (ROC), and inflammatory cytokines reduce the endothelial glycocalyx, thereby directly stimulating the contact between blood circulation substances and endothelium cells, causing structural disorders and proteinuria (39, 40). Increased oxidative stress reduces the production of NO in endothelial cells, and reduced NO availability further leads to endothelial dysfunction (38). Persistent endothelial dysfunction in turn causes changes in vascular tone and permeability, activation of abnormal coagulation function, and abnormal expression of inflammatory cytokines (41).

**Figure 1:**
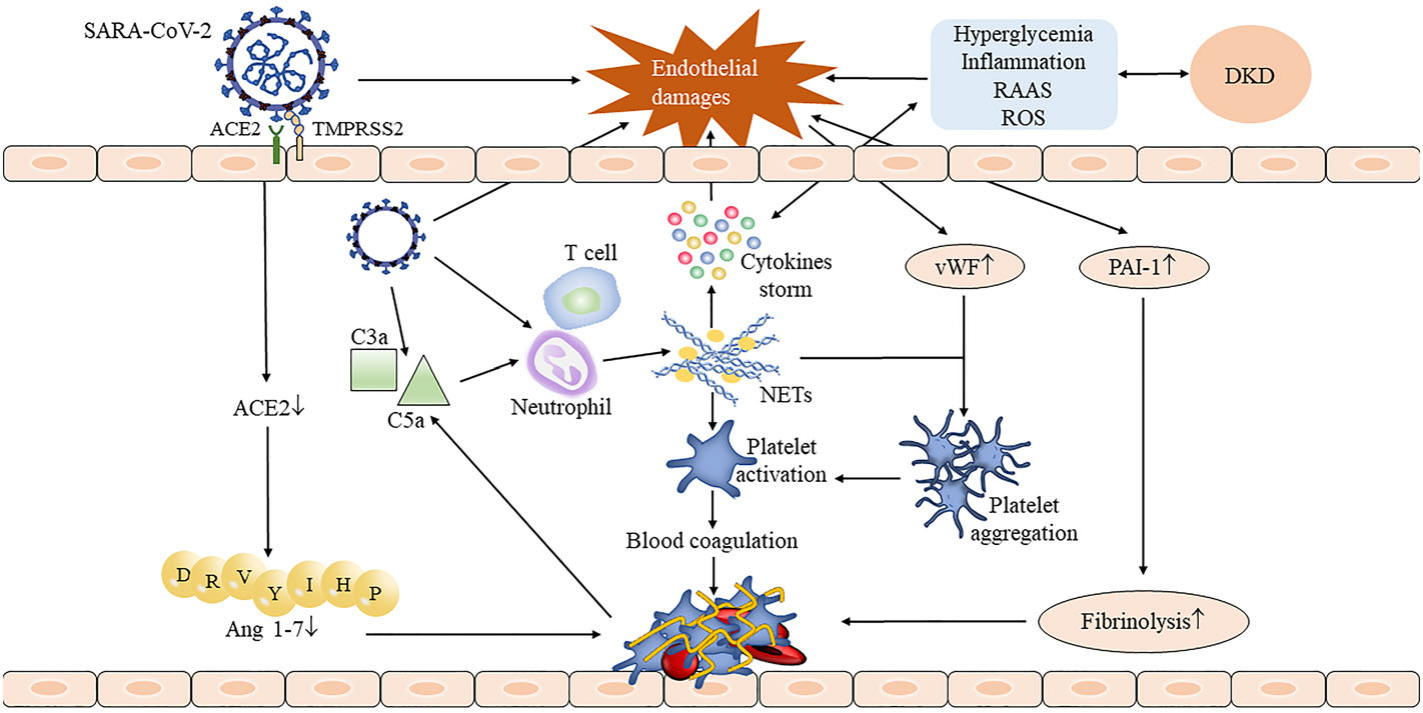
The process of thrombus formation in DKD complicated with COVID-19 patients. The invasion of SARS-CoV-2 damaged the integrity of the endothelium, and the injured endothelial cells stimulated the release of the von Willebrand factor (vWF) and plasminogen activator inhibitor-1 (PAI-1) and accelerated the formation of thrombosis. SARS-CoV-2 activates neutrophils by activating the complement system, thus causing the release of neutrophil extracellular traps (NETs) (48). NETs not only stimulate the occurrence of the coagulation cascade and leads to subsequent thrombosis but also stimulates cytokine storm to further aggravate endothelial damage. Downregulated angiotensin-converting enzyme 2 (ACE2) reduced angiotensin 1-7 production which also contributed to the thrombosis. The presence of DKD may aggravate endothelial damage through inflammatory stimulation, hyperglycemia, and oxidative stress, resulting in a more serious consequence.

DKD-induced endothelial dysfunction further aggravates the serious risk of SARS-CoV-2 infection. Indeed, endothelial dysfunction is also a common feature of COVID-19. Endothelial damage and endothelial cell membrane disruption had been found in COVID-19 autopsies (42). Kidney damage in COVID-19 patients is dominated by elevated creatinine and albuminuria. An excess half of patients of COVID-19 had proteinuria after admission (43, 44). Both direct stimulation of SARS-CoV-2 invasion and indirect stimulation of cytokine storm result in vascular endothelial dysfunction and thus may activate the coagulation cascade (45, 46). Fibrinogen, D-dimer, factor VIII, von Willebrand factor (vWF), and neutrophil extracellular traps (NETs) were greatly increased in COVID-19 model experiment (47) and patients (22); these abnormal coagulation parameters indicating COVID-19 patients are in a hypercoagulable state. On the one hand, SARS-CoV-2 infection activates the complement system to generate pro-inflammatory peptides C3a and C5a and recruit neutrophils to release NETs; it induces thrombosis by activating the blood coagulation contact pathway, which in turn leads to excessive production of thrombin and C5a (46, 48). There seems to be a feedback pathway between the complement system and NETs, which continuously promotes thrombosis (48). On the other hand, NETs also combined vWF secreted by endothelium cells to help adhere and aggregate platelets (46). Damaged endothelial cells increase the release of plasminogen activator inhibitor-1, which inhibits the fibrinolytic system and aggravates thrombosis (48). Moreover, the previously mentioned increase in the ACE/ACE2 ratio was also involved in thrombosis (**Figure 1**). The accumulation of all factors leads to endothelium and vascular damage, which ultimately leads to the occurrence of the acute respiratory syndrome (ARDS) and multiple-organ failure.

The occurrence of COVID-19 with thrombotic events has been shown to have a higher rate of hospitalization and an incidence of ARDS (49) and is independently associated with the risk of death (23). Hertanto et al. reported a patient with a history of T2DM, DKD, and leg pain who was admitted with COVID-19. After the admission, dyspnea improved but the leg pain became progressively worse and computed tomography angiography showed thrombosis of popliteal arteries in both lower limbs (14). This patient has a markedly elevated D-dimer level on admission (14), suggesting that the patient may have been in a hypercoagulable state before admission. After infecting SARS-CoV-2, hyperinflammation, endothelium dysfunction, complement system activation, and hypercoagulable may aggravate limb ischemia, causing secondary harm to the patient. Altogether, endothelial damage and hypercoagulable state are key pathogenic mechanisms of COVID-19, and DKD may stimulate its progression and deterioration.

### Dipeptidyl peptidase 4

Dipeptidyl peptidase 4 (DPP4), also known as CD26, is widely distributed in the kidney, usually present in membrane-bound and soluble forms, involved in the degradation of glucose-dependent insulinotropic polypeptide and glucagon-like peptide-1 (GLP-1), thus interfering with insulin secretion and disrupting glucose homeostasis (50). Tahara et al. noted that the level of DPP4 was correlated with AGEs; elevated AGEs increased the expression of DPP4 in renal tubular cells (51). It is well known that hyperglycemia can activate AGEs and induce subsequent oxidative stress and inflammation response. Various inflammatory cytokines associated with DKD contain truncation sites for DPP4 (52). A cross-sectional study in China shows increased DPP4 activities closely associated with T2DM-related DKD patients, whose oxidation stress, IL-6, and C-reactive protein (CRP) levels increased with the increase in the DPP4 quartile (53).

The DPP4 inhibitor, such as linagliptin and alogliptin, can dramatically mitigate AGE and AGE receptor axis activity, thus alleviating the renal damage caused by inflammation, proteinuria, and oxidative stress in type 1 diabetes (T1DM) and T2DM (54, 55). The elevated concentration of the circulation soluble form of the AGE receptor in diabetics is correlated with the inflammatory marker and proteinuria, which can represent a biomarker for vascular injury in T2DM (56, 57). On the other hand, the DPP4 inhibitor also inhibits the fibrotic pathway mediated by transforming growth factor β (58), which is induced by the interaction between DPP4 and cation-independent mannose-6-phosphate receptor (59). The DPP4 inhibitor has gradually become the first choice treatment for patients with T2DM-related renal failure (60).

**Figure 2:**
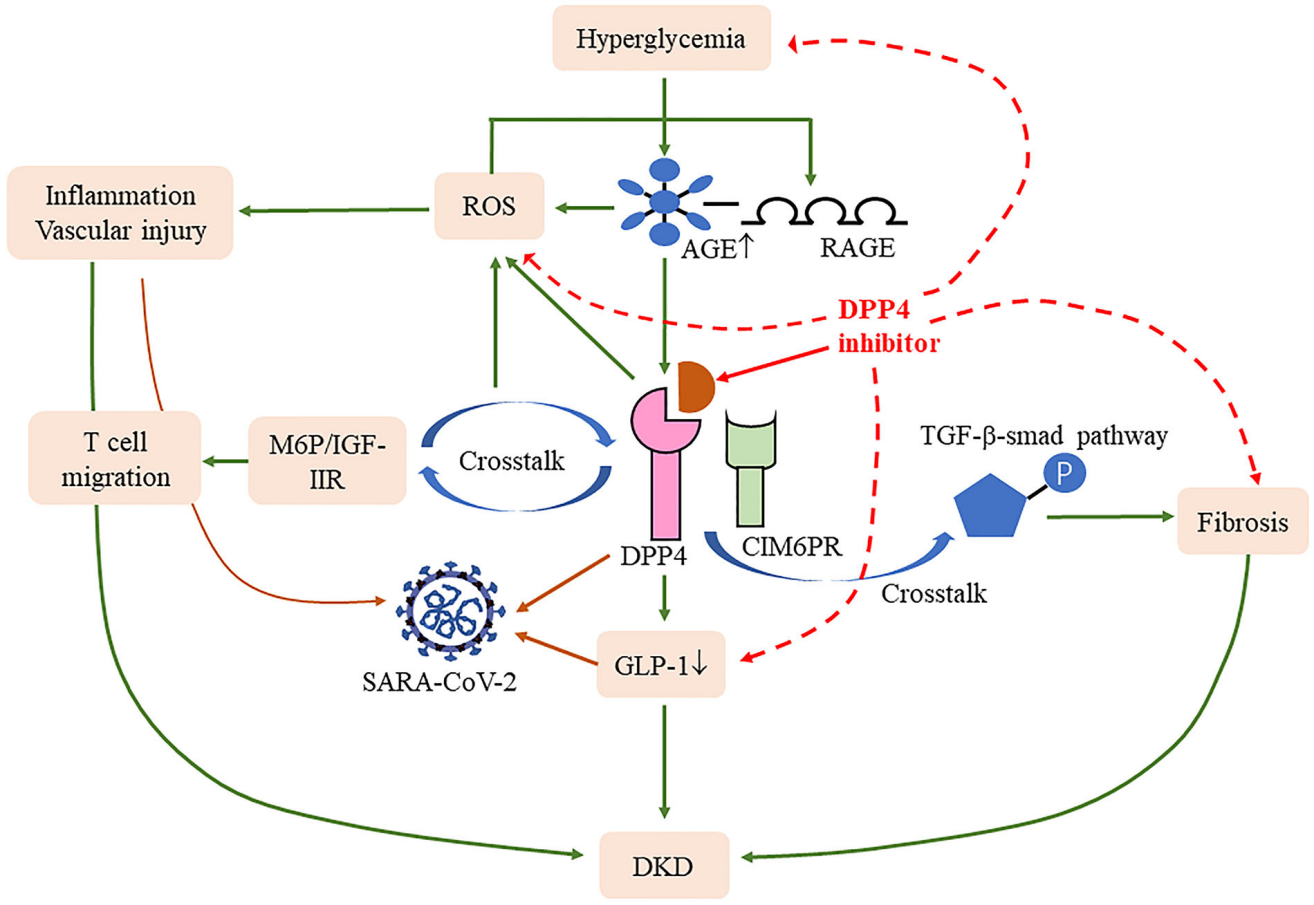
Effects of DPP4 and its inhibitors in COVID-19 and DKD. DPP4 cross talk with D-mannose-6-phosphate/insulin-like growth factor II receptor (M6P/IGF-IIR) (64) and cation-independent mannose-6-phosphate receptor (CIM6PR) (59) induced reactive oxygen species (ROS) and fibrosis, respectively. Meanwhile, DPP4 also induced the degradation of glucagon-like peptide-1 (GLP-1), all of which contribute to the progression of DKD and COVID-19. DPP4 inhibitions blocked these processes and thus improved the disease progression.

A recent paper suggests that the S1 domain of the spike glycoprotein of SARS-CoV-2 may interact with human CD26 to cause disease (61); blocking this connection may reduce the toxic effect of SARS-CoV-2 on the body. It has been well verified in T2DM patients infected with COVID-19, who have mild pneumonia, less non-invasive mechanical ventilation, and better prognosis (62), indicating a range of benefits of the drug for this group. Specifically, DPP4 plays a key role in T-cell activation and immune regulation; it not only modulates CD4+T cell maturation, migration, and cytokine secretion but also interacts with several molecules involved in T-cell function, including mannose 6-phosphate/insulin-like growth factor II receptor, which in turn increased the expression of the AGE receptor through activating reactive oxygen species (63, 64). DPP4 expression is upregulated following T-cell activation (65). Compared with CD28, the mRNA of TNF-α, interferon-γ, and Fas ligand are increased after CD26 co-stimulatory, manifesting greater cytotoxic effects (66). Human T helper type 17 cells also showed a high expression of enzymatically active CD26 (67), while T helper type 17 cells can release IL-17 to recruit monocytes, macrophages, and neutrophils and stimulate cytokines, including IL-1β and IL-6. Solerte et al. reported that T2DM patients infected with COVID-19 using the DPP4 inhibitor had elevated lymphocyte counts, and their CRP and procalcitonin levels were decreased compared to those on conventional treatment (68), suggesting that the anti-inflammatory effect may restore the efficiency of the immune response after SARS-CoV-2 infection. The DPP4 inhibitor also improves bronchoalveolar lavage IL-6 and TNF-α levels in LPS-induced mice and attenuates lung injury (69), meaning direct stimulation of anti-inflammatory activity in the lungs may help ameliorate lung damage from COVID-19. Meanwhile, the decrease in GLP-1 degradation alleviates insulin resistance and its anti-inflammatory can reduce lung injury caused by excessive production of cytokines (50) (**Figure 2**). As can be seen, the therapeutic benefits of DPP4 inhibitors may shed new light for DKD complicated with COVID-19 patients, and more research is warranted to fully evaluate their effect.

### Neuropilin-1

It has been found that neuropilin-1 (NRP-1) mRNA protein is expressed in renal cells and highly located in differentiated podocytes (70, 71). Increased AGEs not only elevate the DPP4 release but also acts on the podocytes. AGE-modified bovine serum albumin downregulated the expression of NRP-1, which may be achieved by reducing the binding ability of the Sp1 transcription factor to the NRP1 promoter to the inhibit the transcriptional activity of the NRP1 promoter in podocytes (71), thus restraining podocyte migration, which leads to increases in “nude” areas of the glomerular basement membrane adhesion to the Bowman space and causes glomerulosclerosis (70). Furthermore, decreased NRP-1 partially inhibited the adhesion capacity of podocytes and impaired cellular reorganization skeleton, which may further influence podocyte injury in DKD and lead to proteinuria (72). Short-term erythropoietin treatment reversed NPR-1 expression, reduced proteinuria, and protected podocytes from AGE-mediated damage (73), which shows that a decreased expression of NPR-1 is one of the characteristics of DKD.

Besides ACE2 as a receptor of SARS-CoV-2 infection, NRP-1 also as an alternative pathway contributes to SARS-CoV-2 infection by binding to the S1 C-end rule motif cleaved by furin protein (74). The role of NPR-1 in COVID-19 has been well described (75). NRP-1 is considered an immune checkpoint for T-cell memory; its immunological role in COVID-19 is unquestionable (75). The Toll-like receptor (TLR) is a key modulator in innate immunity (76). Sultan et al. reported that both expressions of TLR2, TLR4 mRNA, and NRP-1 were elevated in moderate and severe COVID-19 patients, and the NRP-1 level was positively correlated with TLR2 and TLR4 (77). Upregulated NRP-1 is related to deterioration of renal function. This was verified by the significant correlation between NRP-1 expression and serum creatinine and urea levels in severe COVID-19 patients (32, 77). The consumption of NRP-1 after infection may lead to impaired podocyte function and aggravate DKD (78).

Furthermore, NRP-1 contributes to the SARS-CoV-2 infection of pancreatic β-cells and impairs cell function; NRP-1 inhibition can rescue this process (9). Severe COVID-19 may induce blood clotting dysfunction, which leads to the release of various factors from the endothelium. NPR-1 also plays a role in the coagulation process by controlling the adhesion and permeability of endothelial cells (75). Given the above characteristics, severing the combination of the SARS-CoV-2 S1 and NRP-1 could be a new therapeutic target for COVID-19 (79). However, there are few available data on NRP-1 in DKD complicated with COVID-19 patients. Whether the level will be changed in this population remains to be confirmed by further study.

## COVID-19 vaccination

The rapid rollout of vaccines has significantly reduced morbidity and mortality associated with COVID-19 (80). However, associated adverse reactions have been reported; we summarize the cases of hyperglycemia and related complications following COVID-19 vaccination in people with or without diabetes (**Table 2**) (81–97). The majority of cases occur with nausea, polydipsia, and polyuria after 1–4 weeks of COVID-19 vaccination and diagnosed as diabetic ketoacidosis (DKA), hyperosmolar hyperglycemic syndrome, or new-onset diabetes. Of the reported glycated hemoglobin concentrations, those following vaccination were almost higher than baseline; although the exact mechanism is unclear, it may have a similar pathophysiology to the generation of immune and cytokines responses following COVID-19 infection.

**Table 2:** Summary cases of hyperglycemia following COVID-19 vaccination in patients with or without diabetes.

Authors	Country	Age/sex	Medical history	Baseline HbA1c (%)	Vaccine type	Time of symptoms onset	Symptoms	HbA1c (%) after admission	Diagnosis	Treatments after discharge
**Part 1 (Patient with diabetes)**										
Ganakumar et al. (81)	India	20/M	T1DM	ND	Covishield	2 days after second dose	Abdominal pain, decreased appetite, vomit	14.1	DKA	Insulin at a dose of 1.3 units/kg/day
		25/F	T1DM, a history of on and off pain in the right ear	ND	COVAXIN	4 days after second dose	Fever, myalgia, nausea, vomiting, pain abdomen	16.3	DKA, tympanic membrane perforation with acute suppurative otitis media	Insulin at a dose of 1.1 units/kg/day
Lee et al. (82)	America	59/M	T2DM, hypertension, COVID-19 infection 10 months earlier	7.5	Moderna	2 days after first dose	Fatigue, myalgia, subjective fevers, blurry vision, dry mouth, polyuria	13.2	HHS	Metformin 1,000 mg twice daily
		87/M	T2DM, hypertension, hyperlipidemia, ischemic stroke, congestive heart failure, and history of COVID-19 infection	7.0	Moderna	2 days after first dose	Fatigue, myalgias, thirst	ND	HHS, DKA	Metformin 500 mg twice daily
Mishra et al. (83)	India	58/F	T2DM	ND	Covishield	1 day after first dose	ND	ND	Hyperglycemia	Increased dose of metformin
		64/M	T2DM	ND	Covishield	1 days after first dose	Tachycardia, sweat, palpitations	ND	Hyperglycemia	Recovered without additional intervention
		65/M	T2DM	ND	Covishield	6 days after first dose	ND	ND	Hyperglycemia	Recovered without additional intervention
Patrizio et al. (84)	Italy	52/M	T2DM, vitiligo	7	BNT162B2	28 days after second dose	Night fever, weight loss, asthenia	11.5	Graves’ disease, T1DM	Thyroid hormone normal, insulin analogues to reduce glucose
Sakai et al. (85)	Japan	31/F	T1DM, painless thyroiditis	8.3	BNT162b2	1 day after second dose	Sweat, diarrhea, shortness of breath during exertion	8.3	Graves’ disease, hyperglycemia	Thyroid function normalized after 3 months of thiamazole
Yakou et al. (86)	Japan	71/F	Basedow disease, T1DM	8.1	BNT162b2	1 day after first dose	Nausea, fatigue	8.0	DKA	Continue insulin therapy
		52/F	T1DM	10-11.0	BNT162b2	1 day after second dose	Nausea, palpitation and respiratory distress	11.6	DKA	Continue insulin therapy
Zilbermint et al. (87)	America	24/F	T1DM	ND	Moderna	15 h after second dose	Nausea, tachycardia, tachypnea	12.0	DKA	Continue insulin therapy
**Part 2 (patient with non-diabetes)**										
Abu-Rumaileh et al. (88)	America	58/M	Skin tags in his neck	ND	BNT162b2	24 days after first dose	Nocturia, polyuria, polydipsia, severely dehydrated, worse mental status	13	T2DM, HHS	From insulin to metformin
Aydoğan et al. (89)	Turkey	56/M	Vitiligo, Hashimoto’s thyroiditis	5.9	BNT162b2	14 days after second dose	Weight loss, dry mouth, polyuria, polydipsia	8.2	T1DM	Insulin was stopped after 3 months
		48/M	None	5.6	BNT162b2	56 days after second dose	Fatigue, hyperglycemia	10.1	T1DM	Medical nutrition therapy
		27/F	None	ND	BNT162b2	21 days after second dose	blurred vision, polyuria, polydipsia, weight loss, vaginal candidiasis	12.5	T1DM	Discontinued insulin
		36/M	None	ND	BNT162b2	21 days after second dose	Fatigue, dizziness, weight loss, dry mouth	12.6	T1DM	Insulin therapy
Edwards et al. (90)	United Kingdom	59/M	Hypertension, hypercholesterolemia	5.6	Covishield	21 days after first dose	ND	14.1	Hyperglycemic ketosis	ND
		68/M	Hypothyroidism, pre-diabetes	6.5	Covishield	36 days after first dose	ND	14.7	HHS, DKA	ND
		53/M	Hypertension, pre-diabetes	6.2	Covishield	20 days after first dose	ND	17.1	DKA	ND
Lee et al. (82)	America	52/F	Hypertension	5.5-6.2	BNT162b2	2 days after first dose	Polyuria, polydipsia, lightheadedness, dysgeusia	12.0	T2DM, non-ketotic HHS	Metformin 1,000 mg twice daily and weekly dulaglutide 0.75 mg
Makiguchi et al. (91)	Japan	65/F	Lung adenocarcinoma with brain metastases	ND	BNT162b2	ND	Fatigue, appetite loss, and extensive erythema on the trunk	9.4	DKA, T1DM	Insulin therapy
Sakurai et al. (92)	Japan	36/F	None	ND	BNT162b2	3 days after first dose	Thirst, polydipsia, polyuria, palpitations, loss of appetite, fatigue	7.0	DKA, fulminant T1DM	Insulin therapy
Sasaki et al. (93)	Japan	73/F	Osteoporosis, non-tuberculous mycobacterial infection	6-7	Moderna	49 days after second dose	Anorexia, fatigue, nausea and vomit	9.3	T1DM	ND
Sasaki et al. (94)	Japan	45/F	Bronchial asthma	ND	BNT162b2	8 days after first dose	Slight fever, fatigue, thirst, nausea, abdominal pain	7.6	DKA, fulminant T1DM	Insulin therapy
Sato et al. (95)	Japan	43/M	Malignant melanoma	5.4-5.7	mRNA vaccination	2 days after second dose	Thirst, polydipsia, polyuria,	8.0	Fulminant T1DM	Insulin therapy
Tang et al. (96)	China	50/M	None	ND	Inactivated vaccine	5 days after first dose	Polydipsia, polyuria	Near normal	Fulminant T1DM, DKA	Insulin therapy
Yano et al. (97)	Japan	51/F	None	5.6	Moderna	42 days after first dose	Fatigue, thirst, polyuria, polydipsia	10.3	Acute-onset T1DM, DKA	Insulin therapy

Covishield, ChAdOx1 nCoV-19; BNT162b2, Pfizer-BioNTech COVID-19; COVAXIN, BBV152-inactivated whole virion; DKA, diabetic ketoacidosis; F, female; HHS, hyperosmolar hyperglycemic syndrome; M, male; ND, not described; T1DM, type 1 diabetes mellitus; T2DM, type 2 diabetes mellitus.

SARS-CoV-2 invades pancreatic β-cells through ACE2 and NRP-1, thereby destroying insulin secretion, and Müller et al. demonstrated the presence of SARS-CoV-2 antigens in the pancreas (98). Subsequently, downregulated ACE2 elevates the ACE/ACE2 ratio, which in turn activates inflammatory pathways and hypercytokinemia can directly damage the insulin signal; it is reasonable to assume that similar reactions may occur after SARS-CoV-2 antigen presentation following vaccination (90, 99). The impairment of the insulin signal leads to the increase in gluconeogenesis and the decomposition of adipose tissue, which further increases the production of ketone bodies (100) and subsequent hyperglycemia complications. Many cases have reported symptoms of nausea and vomiting after vaccination (101), resulting in patients being unable to consume carbohydrates and history of diabetes-inducing insufficient insulin, which together leads to ketosis; in turn, ketosis aggravates nausea. This vicious cycle leads to the development of DKA in patients (86).

**Figure 3:**
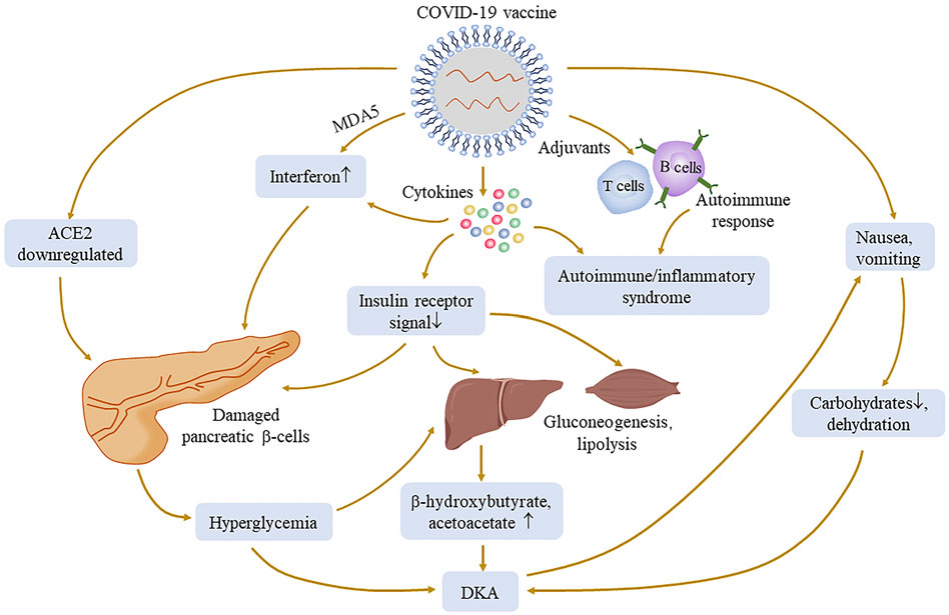
Possible mechanisms of hyperglycemia and its complication associated with COVID-19 vaccination. The injection of COVID-19 vaccines may cause a pathophysiological process similar to that of COVID-19 infection, such as ACE2 downregulation, cytokine production, and immune response. Adjuvants can activate more powerful and lasting specific immune responses to cause autoimmune diseases. The production of cytokines may damage the insulin receptor signal, lead to gluconeogenesis and lipolysis, and increase the substrate concentration of ketone bodies. Melanoma differentiation‐associated protein 5 (MDA5), as an innate pathogen recognition receptor, may interact with vaccines and damage pancreatic β-cells. All of the above processes may cause an abnormal increase of blood glucose.

In the literature, of 16 patients with new-onset diabetes after vaccination, 11 of them were diagnosed with T1DM. Adjuvants are a key component of vaccine by secreting TNF-α, interferon-α, and other cytokines by immune cells to activate more robust and long-lasting specific immune responses, leading to autoimmune/inflammatory syndrome (84, 102, 103). There have been reports of autoimmune diseases caused by COVID-19 vaccination (104). mRNA seems to have self-adjuvant properties that induce immune response. A severe immune response leads to a rapid destruction of cells and severe loss of insulin secretion, which may be one of the causes of T1DM production. Melanoma differentiation‐associated protein 5 (MDA5), as a receptor for recognition of congenital pathogens, induces interferon synthesis, which impairs β-cell insulin production, proinsulin transformation, and mitochondrial function (105) and may recognize RNA of RNA-based COVID-19 vaccines, thus promoting the development of T1DM (92, 106) (**Figure 3**). Meanwhile, for patients with genetic susceptibility, vaccination may aggravate irreversible changes in autoimmune diseases (84, 85). T1DM-related alleles were reported in two patients (94, 97). Therefore, genetically susceptible people need to be more cautious after vaccination. However, it also needs more research to investigate the relationship between T1DM and COVID-19 vaccination. Fortunately, although they need drug treatment, the prognosis was relatively good in all of these cases. Therefore, timely monitoring of blood glucose after vaccination is necessary, regardless of prior diabetes.

## Conclusion

The presence of DKD has been reported to make COVID-19 potentially more severe and fatal, ultimately resulting in ARDS and multiple-organ failure. The relationship between COVID-19 and DKD is complex and bidirectional. RAAS activation, chronic inflammation, and endothelial dysfunction are the core components linking COVID-19 and DKD. Chronic endothelial dysfunction promotes the procoagulant and anti-fibrinolytic state. SARS-COV-2 disrupts the glucose–insulin axis, which increases oxidative stress and exacerbates the progression of DKD. The anti-inflammatory effect of DPP4 inhibitors shows the protective effect on organs. The role of NRP-1 still needs to be further explored. The development of vaccines has greatly suppressed the spread of disease; although there are some adverse reactions, early detection may reduce the harm caused by adverse reactions. Of course, due to the lack of relevant experimental models, more research is needed to illustrate the relationship and prognosis between DKD and COVID-19.

## Author contributions

YY: conducted the data collection and wrote the manuscript. SZ: conducted the data collection and wrote the manuscript. GX: was responsible for the idea and paper revision. All authors contributed to the article and approved the submitted version.

## Funding

The Kidney Disease Engineering Technology Research Centre Foundation of Jiangxi Province (No. 20164BCD40095).

## Conflict of interest

The authors declare that the research was conducted in the absence of any commercial or financial relationships that could be construed as a potential conflict of interest.

## Publisher’s note

All claims expressed in this article are solely those of the authors and do not necessarily represent those of their affiliated organizations, or those of the publisher, the editors and the reviewers. Any product that may be evaluated in this article, or claim that may be made by its manufacturer, is not guaranteed or endorsed by the publisher.

## Abbreviations

ACE2, angiotensin-converting enzyme 2; AGEs, advanced glycation end products; Ang II, angiotensin II; Ang 1-7, angiotensin 1-7; ARDS, acute respiratory syndrome; AT1R, AT1 receptor; COVID-19, coronavirus disease 2019; CRP, C-reactive protein; DKA, diabetic ketoacidosis; DKD, diabetic kidney disease; DPP4, dipeptidyl peptidase 4; GLP-1, glucagon-like peptide-1; HD, hemodialysis; ICU, intensive care unit; IL-6, interleukin-6; MDA5, melanoma differentiation‐associated protein 5; NETs, neutrophil extracellular traps; NO, nitric oxide; NRP-1, neuropilin-1; RAAS, renin–angiotensin–aldosterone system; SARS-CoV-2, severe acute respiratory syndrome coronavirus 2; T1DM, type 1 diabetes; T2DM, type 2 diabetes; TNF-α, tumor necrosis factor-α; vWF, von Willebrand factor.
